# Recent advances in treating oesophageal cancer

**DOI:** 10.12688/f1000research.22926.1

**Published:** 2020-10-01

**Authors:** Kazuto Harada, Jane E. Rogers, Masaaki Iwatsuki, Kohei Yamashita, Hideo Baba, Jaffer A. Ajani

**Affiliations:** 1 Department of Gastrointestinal Medical Oncology, University of Texas M. D. Anderson Cancer Center, 1515 Holcombe Blvd, Houston, TX, 77030, USA; 2 Department of Gastroenterological Surgery, Graduate School of Medical Science, Kumamoto University, 1-1-1 Honjo, Kumamoto, 860-8556, Japan; 3 Department of Pharmacy Clinical Program, University of Texas M. D. Anderson Cancer Center, 1515 Holcombe Blvd, Houston, TX, 77030, USA

**Keywords:** esophageal cancer, esophageal adenocarcinoma, esophageal squamous cell carcinoma

## Abstract

Esophageal cancer (EC) is an aggressive malignancy with an increasing incidence and a poor prognosis. EC is histologically divided into two major categories: adenocarcinoma (EAC) and squamous cell carcinoma (ESCC). EAC and ESCC are molecularly different and therefore treatments should reflect the respective histological subtype. Combined modality therapy is needed for localized EC. When EC is advanced (stage 4), systemic therapy is the mainstay treatment for palliation. For localized EC, several strategies are considered standard, and more trials are necessary to determine a unified and more effective approach. The management for advanced EC is slowly evolving as immunotherapy is showing some promise for ESCC, but more data from ongoing studies are anticipated. Treatment advances will be based on high-definition genomic investigation of individual tumors. Herein, we review the contemporary trends in diagnosing and treating EAC and ESCC.

## Introduction

Esophageal cancer (EC) is the eleventh most common cause of cancer worldwide (473,000 cases) and the sixth most common cause of cancer-related mortality (436,000 deaths) ^1^. ECs are classified into two common histologic subtypes: adenocarcinoma (EAC) and squamous cell carcinoma (ESCC). ESCC is prevalent in Eastern Europe and Asia (endemic areas) but has become less common in Western countries. EAC is prevalent in North America and Western Europe. Tobacco and alcohol consumption and ALDH2 heterozygosity are the major risk factors for ESCC ^2, 3^, while obesity and Barrett’s esophagus are the major risk factors for EAC ^4, 5^. EAC and ESCC are molecularly different, thus their treatments should be different. However, there is still considerable overlap between EAC and ESCC treatment. Thus, the need to differentiate treatment based on histology should be one of the goals of future research ^6^.

## Treatment for resectable EAC

### Endoscopic resection

The European Society of Gastrointestinal Endoscopy and The National Comprehensive Cancer Network (NCCN) guidelines have recommended that an endoscopic resection is indicated for patients with T1a or superficial pT1b tumors that are ≤3 cm in tumor diameter, do not have clear lymphovascular invasion (LVI), and do not have poorly differentiated histology ^7, 8^. A recent Japanese study has provided a similar indication ^9, 10^. However, long-term outcomes after endoscopic resection remain controversial for pT1b tumors. Endoscopic submucosal dissection (ESD) is preferred over a piecemeal resection for accurate pathological evaluation. Endoscopic mucosal resection (EMR) has the limitation of size for an en bloc resection, generally tumors of 1.5 to 2 cm in diameter. An ESD requires higher endoscopic skill, but it enables en bloc resection regardless of the tumor size. A small randomized study of high-grade dysplasia or early EAC in which tumor size was 3 cm or smaller in diameter showed that ESD provided higher R0 and curative resection rates than EMR ^11^. However, it remains unclear whether ESD improves long-term outcomes.

### Combined modality therapy

Surgery is the most effective strategy to cure localized EAC in early stage disease. However, surgery alone is usually inadequate in advanced cases. Preoperative chemoradiation or perioperative chemotherapy are currently utilized as an adjunct to surgery. The choice of preoperative strategy is largely based on practice preferences.

The CROSS trial provided evidence for the efficacy of preoperative chemoradiation followed by surgery over surgery alone ^12^. EC patients were randomly assigned to one of two groups: preoperative chemoradiation (n = 180) and surgery alone (n = 188). Patients were highly selected. The overall survival (OS) for the preoperative chemoradiation group was significantly longer than that for the surgery alone group (median OS 48.6 months vs. 24.0 months; hazard ratio [HR] 0.68; 95% confidence interval [CI] 0.53–0.88; *P* = 0.003) ^13^. Another randomized trial (CALGB 9781) assessed only 56 patients but also showed benefit for preoperative chemoradiation ^14^. However, the benefit from preoperative chemoradiation for patients with EAC with stage I/II EC remains debatable based on the result of the FFCD 9901 trial, which showed similar R0 resection rate and no OS benefit but increased postoperative mortality ^15^.

The MAGIC trial was designed to investigate chemotherapy in gastric cancer. The study cohort also contained a proportion of patients with gastroesophageal and proximal gastric cancers. This study found that the perioperative chemotherapy group (three preoperative and three postoperative cycles of epirubicin, cisplatin, and fluorouracil [ECF]) had a favorable OS compared with surgery alone (5-year rate: 36% vs. 23%; HR 0.75; 95% CI 0.60–0.93; *P* = 0.009) ^16^. Following this trial, especially in Europe, the MAGIC regimen was the preferred standard, but the use of epirubicin had been controversial ^17^. Recently, the FLOT4 trial showed survival benefit of 5-fluorouracil, leucovorin, oxaliplatin, and docetaxel (FLOT) over ECF ^18^. A total of 716 patients were randomized to receive FLOT (n = 356) or ECF (n = 360). Median OS was significantly longer in the FLOT group compared with the ECF group (median OS 50 months vs. 35 months; HR 0.77; 95% CI 0.63–0.94). Although the reported rate of pathological complete response (pCR) was higher in the FLOT group than in the ECF group (16% vs. 6%; *P* = 0.02), this conclusion remains unclear ^18^. Adverse events were more frequent in the FLOT group. The most notable adverse effect differences were the incidence of grade 3 or 4 infections (18% vs. 9%), neutropenia (51% vs. 39%), diarrhea (10% vs. 4%), and neuropathy (7% vs. 2%). The number of patients with severe adverse events, including those occurring during the hospital stay for surgery, were similar in the two groups: 27% in the ECF group and 27% in the FLOT group. Given the toxicity profile, the FLOT regimen should be reserved for patients with a good performance status. The difficulty of perioperative chemotherapy, particularly in completion of postoperative chemotherapy, remains an issue, as seen by the FLOT4 trial. FLOT4 showed that, even in relatively healthy patients enrolled in a clinical trial, the number who completed all allocated cycles was low: 37% in the ECF group and 46% in the FLOT group. Combination therapy with two cytotoxic drugs, for example oxaliplatin plus 5-fluorouracil (FOLFOX), is considered a perioperative recommended regimen for patients who have good to moderate performance status ^19– 22^. Overall, the projected 5-year survival rate of 40–45% emphasizes a need for significant improvement in the treatment of these cancers.

R0 resection is one of the most important factors for selecting surgery. R0 resection rate is worse in Siewert I or primary EAC than gastric cancer, even after preoperative chemotherapy ^23^. This result suggests that local control by adding radiation could be needed for primary EAC. It should be noted that the FLOT4 trial included many patients with gastric cancer.

## Systemic therapy for metastatic EAC

### First-line systemic therapy

Systemic therapy for metastatic EAC has been based on a study designed together with gastric adenocarcinomas. Fluoropyrimidines (5-fluorouracil or capecitabine) combined with either oxaliplatin or cisplatin has been the global standard therapy for decades ^24^. For patients with HER2-positive EAC, adding trastuzumab to fluoropyrimidine plus platinum is recommended based on the ToGA trial ^25^. Other molecular targeted drugs were assessed, but no additional targeted agents were found to be beneficial for EAC as the first-line therapy at this point in time ^24^.

### Second-line or subsequent systemic therapy

Ramucirumab plus paclitaxel is the preferred regimen for second-line therapy based on the RAINBOW study ^26^. Ramucirumab monotherapy is an option for patients who are not candidates for combination therapy with paclitaxel ^27^. Single administration of chemotherapeutic agents, such as irinotecan and taxanes, significantly improves OS compared with best supportive care ^28^.

Assessment of microsatellite instability (MSI) status and programmed death ligand 1 (PD-L1) expression is recommended. However, MSI tumors are rare in EC, and PD-1 or PD-L1 blockade is marginally effective in EAC. Recently, based on several trials, such as KEYNOTE-016, KEYNOTE-164, KEYNOTE-012, KEYNOTE-028, and KEYNOTE-158, pembrolizumab has been approved as a second-line regimen for patients with MSI-high/deficient mismatch repair (MMR) solid tumors, regardless of the tumor type. Le *et al.* reported that objective radiographic response (ORR) was observed in 53% of patients, and complete response was achieved in 21% of patients with deficient MMR tumor ^29^. The KEYNOTE-059 study showed that ORR was as high as 57% in patients with MSI-high tumors, which is significantly higher than 9% in the case of microsatellite stable tumors ^30^.

In the United States in 2017, pembrolizumab was approved for advanced EAC patients with PD-L1-positive tumors (combined positive score [CPS] >1) who have cancer progression after two or more prior therapies. In the KEYNOTE-059 trial, 259 patients with gastric or esophagogastric junction (EGJ) adenocarcinoma who have progressed after two or more prior therapies were assessed. Pembrolizumab monotherapy showed that ORR was higher in patients with PD-L1-positive disease than in those with PD-L1-negative disease (15.5% vs. 6.4%) ^30^.

ATTRACTION-2 evaluated the efficacy of nivolumab in patients with advanced gastric or EGJ adenocarcinoma who underwent at least two previous chemotherapy regimens. Here, 493 patients were randomly assigned to receive nivolumab (n = 330) or placebo (n = 163) ^31^. Median OS was 5.26 months in the nivolumab group and 4.14 months in the placebo group (HR 0.63; 95% CI 0.51–0.78; *P* <0.001). Moreover, the survival benefit with nivolumab was independent of PD-L1 expression. Thus, nivolumab is accepted as third-line therapy regardless of PD-L1 expression in Japan.

Recently, the phase III TAGS trial evaluated the efficacy of TAS-102, an orally administered combination of a thymidine-based nucleic acid analogue, trifluridine, and a thymidine phosphorylase inhibitor, tipiracil hydrochloride, in metastatic gastric and EGJ adenocarcinoma as third-line therapy ^32^. A total of 507 patients were randomly assigned to the trifluridine/tipiracil group (n = 337) and to the placebo group (n = 170). Median OS was 5.7 months in the trifluridine/tipiracil group and 3.6 months in the placebo group (HR 0.69; 95% CI 0.56–0.85; *P* = 0.00058) ^32^. Thus, TAS-102 was approved as an option for third-line therapy. However, only a select population might be suitable for TAS-102 because of the lack of response rate.

## Treatment for resectable ESCC

### Endoscopic resection

The NCCN guidelines state that endoscopic resection is adapted for early stage disease (pTis, pT1a, selected superficial pT1b without LVI and favorable histology) ^33^. Japanese esophageal cancer guidelines recommend endoscopic resection for only T1a ESCC tumors ^34, 35^. Lymph node metastases of pT1a-epithelium (EP)/lamina propria mucosae (LPM) is very rare, but pT1a- muscularis mucosae (MM) can develop metastases, in some cases in 10% of patients ^36^. T1b tumors are generally not considered appropriate for endoscopic treatment in Japan ^37^. Recently, the Japan Clinical Oncology Group (JCOG) 0508 trial suggested that endoscopic resection in combination with chemoradiation is efficacious for cT1bN0M0 ESCC as an esophagus-preserving treatment ^38^.

### Combined modality therapy

ESCC is very sensitive to chemoradiation, and this results in higher rates of complete tumor regression and better local tumor control, but it is unclear if a better survival is achieved than in EAC patients ^39^. Thus, preoperative chemoradiation is globally recommended as standard of care for treating ESCC. Moreover, definitive chemoradiation is an acceptable option for treating ESCC. The FFCD 9102 trial found that, for ESCC patients who respond to chemoradiation, adding surgery had no survival benefit compared with the continuation of additional chemoradiation ^40^.

Preoperative chemotherapy is also an option for ESCC. The OEO2 trial showed that the perioperative chemotherapy (two cycles of FP; cisplatin and fluorouracil) group had a favorable OS compared with the surgery alone group (5-year OS rate: 23% vs. 17%; HR 0.84; 95% CI 0.72–0.98; *P* = 0.03), but the rate of ESCC patients in this study was only 34% ^20, 21^. The JCOG9907 trial compared preoperative and postoperative chemotherapy (two cycles of FP) for stage II or III ESCC ^41^. OS in the preoperative group was better than in the postoperative chemotherapy group, but a limitation for this protocol was that patients with pN0 in the postoperative chemotherapy group did not undergo chemotherapy.

## Systemic therapy for advanced ESCC

### First-line systemic therapy

As with EAC, fluoropyrimidine (5-fluorouracil or capecitabine) combined with either oxaliplatin or cisplatin has been the most commonly used first-line regimen for advanced ESCC ^42^. No targeted therapy has proven effective in this disease to date ^43^.

### Second-line or subsequent systemic therapy

Single-agent chemotherapy with taxanes or irinotecan is an option for second-line therapy ^44– 46^. However, results with second-line chemotherapy in ESCC are inferior to those in EAC.

An immune checkpoint inhibitor has been approved as second-line or subsequent therapy for advanced ESCC. Pembrolizumab has also been accepted as a second-line therapy for patients with advanced ESSC with PD-L1 expression levels by CPS of >10. The phase III KEYNOTE-181 trial compared pembrolizumab versus investigator’s choice chemotherapy (docetaxel, paclitaxel, or irinotecan) as second-line therapy in 628 patients with advanced EC ^47^. Pembrolizumab significantly improved median OS (9.3 months vs. 6.7 months; HR 0.69; 95% CI 0.52–0.93; *P* = 0.0074) and 12-month OS rates (43% vs. 20%) compared with chemotherapy in patients whose tumors had a PD-L1 CPS >10.

Recently, nivolumab has been accepted as a second-line therapy for ESCC in Japan based on ATTRACTION-3 outcome ^48^. A total of 419 previously treated patients with ESCC were randomly assigned to nivolumab (n = 210) and chemotherapy (n = 209). OS was significantly improved in nivolumab; median OS in the nivolumab and chemotherapy group was 10.9 months and 8.4 months, respectively (HR 0.77; 95% CI 0.62–0.96; *P* = 0.019) ^48^. In the KEYNOTE-180 trial, 121 patients with EC (63 ESCC and 58 EAC) who progressed after two or more prior therapies were assessed. Pembrolizumab monotherapy showed that ORR was 14.3% (95% CI 6.7–25.4%) in patients with ESCC and 5.2% (95% CI 1.1–14.4%) in patients with EAC ^49^. ORR was higher in patients with PD-L1-positive tumor (13.8% vs. 6.3%) ^49^. These results demonstrated the efficacy and tolerability of pembrolizumab as a third-line or subsequent therapy option in patients with heavily pretreated ESSC with high PD-L1 expression.

## Future perspective

### Preoperative chemoradiation or chemotherapy

Preoperative chemotherapy and preoperative chemoradiation strategies remain popular in different regions of the globe. A recent meta-analysis using network meta-analyses showed better OS in preoperative chemoradiation than in preoperative chemotherapy (HR 0.83; 95% CI 0.70–0.96) ^50^. A retrospective review from our institution showed that preoperative chemoradiation was associated with a longer OS and a higher pCR rate in EAC ^51^. To date, three randomized trials have compared preoperative chemoradiotherapy and chemotherapy, but none could reach a definitive conclusion ^52–54^. The following studies are comparing preoperative chemoradiation and chemotherapy: the PROTECT study (NCT02359968), Neo-AEGIS trial (NCT01726452), and NEOSCOPE trial (NCT01843829). In Japan, a three-arm phase III trial (JCOG1109) is comparing preoperative FP versus preoperative DCF versus preoperative chemoradiation for ESCC ^55^. These ongoing trials may provide considerable information on treatment options in the future.

### Personalized therapy for esophagus preservation

Approximately 23% of patients with EAC and 49% of patients with ESCC achieve a pCR after preoperative chemoradiation ^12^. However, in other datasets, pCR rates are no better than 20%. One future hope is to avoid esophagectomy in select patients who are destined to achieve a pCR. However, a clinically implementable strategy to achieve this is currently lacking.

In EAC, we have reported that one positron emission tomographic (PET) parameter, total lesion glycolysis, could help to identify a population who can be cured after definitive chemoradiation ^56^. In addition, we reported that PET responses might predict survival and pathological response for chemoradiation ^57^. Thus, a strategy of selective surgery for remnant tumor might contribute to preserving the esophagus, but it still remains a challenge ^58^. Accurate detection of residual tumor is difficult. The preSANO trial assessed optimal clinical response evaluation. Here, it was concluded that endoscopic ultrasonography with bite-on-bite biopsies and fine-needle aspiration of suspicious lymph nodes were suitable for the detection of locoregional residual disease (clinically false negative cases 10% in tumor regression grade 3 or 4) and PET-CT can be used for the detection of interval metastases ^59^. Selective surgery by active surveillance is currently being assessed in the SANO trial ^60^.

Because ESCC is more responsive to chemoradiation than EAC, preserving a patient’s esophagus may be more feasible. The JCOG0502 trial compared survival for definitive chemoradiation and esophagectomy in stage I ESCC and demonstrated that the survival of definitive chemoradiation was non-inferior against that of an esophagectomy ^61^. However, in the JCOG0502 trial, salvage esophagectomy was performed in 21 of 159 patients (13.2%) in the chemoradiation group, and it should be noted that morbidity following salvage esophagectomy is high. A retrospective review from our institution showed that major postoperative complications after salvage esophagectomy frequently occurred in 71.4% ^62^. The JCOG1406-A trial compared two Japanese trials conducted in the same era: JCOG9906, which evaluated definitive chemoradiation, and JCOG9907, which evaluated preoperative chemotherapy followed by esophagectomy for clinical stage II/III ESCC ^63^. Although it is not a direct comparison, OS was better in the preoperative chemotherapy followed by esophagectomy group than in the definitive chemoradiation group (HR 1.72; 95% CI 1.19–2.50) ^63^. These data suggest that esophagectomy might be superior to definitive chemoradiation for clinical stage II/III ESCC, but a head-to-head comparison is lacking.

### Molecular biology

TCGA reported an integrated genomic landscape in EAC and ESCC ^6, 64^. However, there are few studies assessing the association of genomic subtype and therapeutic response. We sequenced the whole exome and transcriptome of peritoneal metastatic cells and demonstrated distinct genomic alterations compared with primary tumor cells, suggesting that treatment strategy should be based on not only the cancer type but also the metastatic site ^65^. Recently, liquid biopsy, such as circulating tumor cells, cell-free DNA, and exosomes, has the potential to predict early treatment response or identify intratumoral heterogeneity ^66– 68^. Further studies and clinical applications are expected. Therefore, in-depth study of the tumor’s biology is warranted and may contribute to personalized therapy.

## Conclusion

We have described the current understanding of the treatment options for EAC and ESCC. Combined modality therapy is needed for resectable EC. Head-to-head comparisons of preoperative chemotherapy and chemoradiation are warranted. The management for advanced EC has been evolving partly owing to the use of immune checkpoint inhibitors. The future challenge is to identify molecular targets based on tumor profiling.
